# Genome-Wide Association Study Identifies Genetic Associations with Perceived Age

**DOI:** 10.1016/j.jid.2020.03.970

**Published:** 2020-12

**Authors:** Victoria Roberts, Barry Main, Nicholas J. Timpson, Simon Haworth

**Affiliations:** 1 Bristol Medical School, University of Bristol, Bristol, United Kingdom; 2 Bristol Dental School, University of Bristol, Bristol, United Kingdom; 3 MRC Integrative Epidemiology Unit, Department of Population Health Sciences, Bristol Medical School, University of Bristol, Bristol, United Kingdom

**Keywords:** BMI, body mass index, FDR, false discovery rate, GCT-VL, Complex Trait Genomics Virtual Lab, GWAS, genome-wide association study, LD, linkage disequilibrium, MAF, minor allele frequency, SNV, single nucleotide variant

## Abstract

Failure of dermal protection or repair mechanisms might lead to visibly aged skin. The study aimed to identify genetic associations with perceived age. A genome-wide association study was undertaken in 423,992 adult participants of UK Biobank, using questionnaire data on perceived age and genetic data imputed to the Haplotype Reference Consortium imputation panel. The study identified 74 independently associated genetic loci, to our knowledge previously unreported (*P* < 5 × 10^−8^), which were enriched for cell signaling pathways, including the *NEK6* and *SMAD2* subnetworks. Common genetic variation was estimated to account for 14% of variation in perceived age, and the heritability of perceived age was partially shared with that of 75 other traits, including multiple traits representing adiposity, suggesting that perceived age may be a useful proxy trait in genetic association studies.

## Introduction

Skin is the interface between the internal and external environment and has functions to prevent and repair damage from exogenous factors such as UV light and bacteria (Lee et al., 2006). Studies that characterize the biological mechanisms underlying normal skin barrier and repair functions may provide insight into diseases where these mechanisms fail (Williams, 2005), such as atopy; proxy phenotypes for skin response to exogenous factors; and improve understanding of these mechanisms.

One challenge is the ability of skin to respond to UV light, which has been explored using genome-wide association studies (GWASs) for proxy phenotypes such as self-reported tanning ability (Nan et al., 2009, Visconti et al., 2018), identifying a range of associated loci. Objective or subjective measures of skin age might represent the ability of skin to respond to a wider range of environmental challenges. Studies have investigated genetic factors associated with skin age, rationalizing that failure of photoprotective or other dermal integrity mechanisms will lead to visibly aged skin (Law et al., 2017, Liu et al., 2016). The results of these studies and GWASs investigating self-reported tanning identified association at the *MC1R* gene, which regulates pigmentation. Other genes identified have been linked to skin and hair pigmentation, poor tanning ability, increased freckling, and skin cancers (Duffy et al., 2010, Duffy et al., 2004, Han et al., 2011, Han et al., 2008, Kita and Fraser, 2016, López et al., 2014, Sulem et al., 2007, Zhang et al., 2013). This apparent similarity in findings for objective and subjective measures of skin function is mirrored in studies where multiple measures of skin appearance and function are available in the same participants (Oyetakin-White et al., 2015).

We reason that subjective measures of perceived age may act as a proxy for underlying dermal integrity and photoprotective mechanisms. This study aims to characterize genetic associations with perceived age, focusing on understanding heritability; identify genetic loci; and understand whether genetic mechanisms regulating dermal integrity are shared with other risk factors or diseases.

## Results

### Participants

A GWAS was performed for perceived age. After final exclusions, analysis included 423,992 adult participants. Of these, 8,630 reported looking older than their biological age, 103,300 reported looking about their age, and 312,062 reported looking younger than their biological age. There were trends with both age and sex, where females were more likely to report looking young for their age than males, and older participants were more likely to report looking young for their age than young participants (Supplementary Table S1). As there was an imbalance of responses in these three groups, the effective statistical power of the experiment was smaller than the total sample size (see Supplementary Text S1 for an estimate of effective sample size).

### Total heritable contribution

After final quality control, approximately 9.6 million single nucleotide variants (SNVs) with a minor allele frequency (MAF) of 0.1% or greater were tested for association. There was evidence for inflation in test statistics (genomic control factor = 1.49), which is typical for large studies of complex, polygenic traits and lower than that reported in recent studies of height and body mass index (BMI) (Yengo et al., 2018). Linkage disequilibrium (LD) score regression analysis estimated that 14% (SE, 0.6%) of variation in perceived age was due to the effects of common genetic variants and that polygenic heritability rather than inflationary bias was responsible for most of the inflation in genomic control factor (LD score regression ratio = 0.09).

### Single variant findings

**Figure 1:**
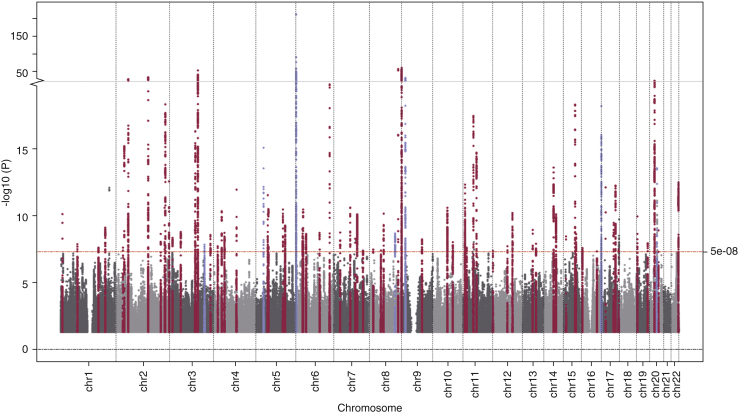
**Manhattan plot of GWAS.** Genomic regions independently meeting genome-wide significance are highlighted in magenta (to our knowledge previously unreported loci) or blue (positive controls). The red line at *P* = 5 × 10^−8^ indicates the conventional threshold for genome-wide significance. The y-axis scale is split and truncated at *P* = 1 × 10^−250^. GWAS, genome-wide association study.

There was evidence for association at 5,395 SNVs representing 81 independent signals of association (*P* < 5 × 10^−8^). Of those, 74 represented, to our knowledge, previously unreported discoveries, whereas seven were in the region of loci previously reported for a skin appearance– or facial age–related trait (Figure 1). A subset of lead variants is presented in Table 1, and full results are provided in Supplementary Table S2.

**Table 1:** Top 10 Independently Associated Lead Variants in GWAS

Chr	SNP	Position	Effect Allele	EAF	Beta	SE	OR	Locus	*P* (Conditional)
9	rs520015	211762	C	0.51	0.070	0.0043	1.07	*C9orf66* *- DOCK8*	1.2 × 10^−58^
8	rs10956486	130699140	T	0.68	−0.073	0.0046	0.93	*GSDMC*	2.6 × 10^−55^
3	rs61263161	126691104	G	0.83	−0.087	0.0058	0.92	*CHCHD6*	1.4 × 10^−51^
2	rs1438898	145714354	A	0.75	0.059	0.0050	1.06	*AC074093.1*	3.5 × 10^−32^
2	rs76032374	56058356	A	0.87	0.068	0.0064	1.07	*EFEMP1*	7.9 × 10^−27^
20	rs28897169	22100542	T	0.39	0.043	0.0044	1.04	*LOC100270679*	7.0 × 10^−22^
6	rs4869723	151579432	C	0.56	−0.040	0.0044	0.96	*AKAP12*	1.7 × 10^−20^
2	rs116254882	223025055	G	0.96	0.095	0.0107	1.10	*PAX3*	5.2 × 10^−19^
15	rs1550436	74221157	C	0.53	−0.039	0.0043	0.96	*LOXL1*	5.5 × 10^−19^
2	rs7590866	223087329	G	0.86	−0.054	0.0062	0.95	*PAX3*	2.3 × 10^−18^

Abbreviations: Chr, chromosome; EAF, effect allele frequency; GWAS, genome-wide association study; SNP, single nucleotide polymorphism; SNV, single nucleotide variant.

Each row contains a lead SNV representing an independent signal of association following a stepwise selection procedure with *P* < 5.0 × 10^−8^ after conditioning on previously reported signals of association. The position column contains genomic position based on build 37 (GRCh37.p13) of the human genome. The beta coefficient and accompanying SE are on a log-odds scale and have been exponentiated to provide an odds ratio for reference. ORs > 1 reflect increased odds of appearing young. The locus column includes the name of the gene nearest the lead SNV. Results for all independent lead variants are provided in Supplementary Table S2 with both conditional and unconditional *P*-values. Full results for all variants are provided as a link in the data access statement.

Of these genetic loci, the strongest statistical evidence was seen at *C9orf66-DOCK8* with a lead signal carried by rs520015_C (effect allele frequency, 0.51; OR, 1.07; *P* = 1.0 × 10^−58^). Single nucleotide polymorphisms were mapped to genes using positional mapping tools in FUMA (Watanabe et al., 2017). Rs520015 was annotated as an intergenic variant in LD with a missense variant within *C9orf66* (rs481905, r^2^ = 0.81) and multiple intronic variants within *DOCK8* (e.g., rs2484966, r^2^ = 0.88).

Of the genetic loci discovered, the largest effect size of a minor allele was estimated for rs139356332_G (effect allele frequency, 0.98; OR, 0.90; *P* = 8.1 × 10^−13^), an uncommon intronic variant within *MFAP4.* This gene encodes an extracellular glycoprotein that is thought to contribute to the organization of elastin fiber components in the extracellular matrix (Pilecki et al., 2016).

The study replicated evidence for association at seven previously studied loci, with the strongest evidence at rs12203592_C, a common intronic variant within *IRF4* with large effects on odds of appearing youthful (effect allele frequency, 0.78; OR, 1.22; *P* = 1.2 × 10^−327^). This locus has been reported for skin age–related traits, including pigmented spot severity, perceived skin aging, tanning ability, risk of sunburn, and tanning response to sun (Jacobs et al., 2015, Law et al., 2017, Visconti et al., 2018, Zhang et al., 2013).

### In silico transcriptome-wide association analysis and fine mapping

The predicted expression of 25,812 gene transcripts was tested for association with perceived facial age. A total of 175 gene transcripts passed a Bonferroni-corrected *P*-value threshold (*P* < 1.9 × 10^−6^). There was inflation in these results (Supplementary Figure S1), which might represent polygenic association signal but could also be related to correlation in the predicted expression of adjacent genes seen using the S-PrediXcan method (Wainberg et al., 2019). There was high concordance with the results of single variant analysis, and nearly all transcripts were in genomic loci already highlighted by the single variant results. The strongest evidence for association was *IRF4*, where higher transcription was predicted to associate with lower odds of looking youthful (*P* = 1.6 × 10^−77^) (Supplementary Table S3). The FOCUS method (Mancuso et al., 2019) for probabilistic fine mapping was applied to help resolve correlation in adjacent predictions and nominate a credible set of biologically causal genes underlying this association signal. This was able to resolve some association signals with a high degree of confidence; for example, an association signal on chromosome 2 represented by rs1438898 in single variant results that mapped to a uncharacterized transcript *AC074093.1* using positional mapping was mapped to *ZEB2* with a high probability using fine mapping. Conversely, the fine mapping approach was unable to produce stable models for associated loci on chromosome 6, and results from this chromosome are not presented. The single gene with the highest posterior probability estimate for each locus is reported in Supplementary Table S4, and full results are included in Supplementary Table S5.

### Enrichment in gene sets and tissue expression

Enrichment analysis was performed using DEPICT (Pers et al., 2015) implemented in the Complex Trait Genomics Virtual Lab (GCT-VL) (G Cuéllar-Partida, unpublished data). Analysis identified enrichment (false discovery rate [FDR] ≤ 0.05) in 23 predefined sets of functionally related genes, with the strongest enrichment signal seen for the *NEK6* subnetwork (*P* = 3.2 × 10^−7^) (Supplementary Table S6). Enrichment in tissue-specific transcription in 209 tissues was tested but did not identify any enrichment beyond chance (*P*_*FDR*_ > 0.05 for all tissues) (Supplementary Table S7).

### Shared heritability and genetic causality proportions

Genetic correlation with 1,362 traits was estimated, of which 75 traits had evidence for shared heritability with perceived age after a Benjamini-Hochberg correction for multiple testing (*P*_*FDR*_ < 0.05). All genetic correlation estimates lay within the range −0.5 to +0.5, suggesting that the genetic determinants of perceived age are not fully captured by other traits in the GCT-VL (G Cuéllar-Partida, unpublished data) catalog.

The strongest evidence for genetic correlation was seen with obesity-related traits, where genotypes that associated with greater adiposity overlapped with genotypes associated with reduced odds of appearing youthful (e.g., BMI: genetic correlation = –0.25, *P*_*FDR*_ = 2.9 × 10^−12^ and waist circumference: genetic correlation = –0.23, *P*_*FDR*_ = 2.3 × 10^–13^) (Supplementary Table S8).

These correlations might be due to shared genetic influences on perceived age but may be due to vertically pleiotropic pathway effects where one trait exerts a causal effect on the other. To investigate this further, genetic causality proportions (O’Connor and Price, 2018) were estimated for all pairs of traits with a significant genetic correlation using an online platform (S Haworth, unpublished data). The single trait with the strongest evidence for causal effect was BMI. The effects of greater BMI on reduced odds of appearing youthful were modeled to explain part of the genetic correlation with BMI (genetic causality proportion estimate = –0.64); however, neither this finding (*P*_*FDR*_ = 0.07) nor any other causality proportions were considered significant after correction for multiple testing (Supplementary Table S9).

## Discussion

This study used genome-wide analysis to investigate genetic contributions to perceived age using the rationale that failure of dermal repair mechanisms would lead to visibly aged skin. There was evidence for a polygenic heritable contribution to youthful appearance, and single variant analysis identified 74 loci to our knowledge previously unreported. These loci were enriched for gene sets encoding a range of regulatory networks, supporting the idea that a range of different biological processes are implicated in maintaining a youthful appearance. The *NEK6* subnetwork, identified in gene set enrichment analysis, helps govern the initiation of mitosis and progression through the cell cycle and prevents cell senescence (Jee et al., 2010). We hypothesize that natural variation in genes encoding the *NEK6* subnetwork leads to variation in the ability to compensate for age-related decline in tissues, resulting in variation in signs of aging. This is supported by enrichment in other regulatory networks with roles in growth signaling, including the *SMAD2*, *SMAD4*, and *SMAD9* subnetworks. This mechanism is likely one of several diverse pathways that contribute to maintaining a youthful appearance; for example, we also observed enrichment in lipid-mediated signaling.

Although perceived age may capture variation in skin biology and response to environmental challenges, it is likely to also capture other biological factors such as nutritional status. To help investigate the degree of overlap with other traits and biological specificity of the phenotype, we estimated genetic correlations with traits and diseases in a hypothesis-free manner and identified genetic correlations with 75 other traits or diseases. These correlations were modest in magnitude, suggesting that perceived age provides a proxy for an underlying phenotype which has not been extensively explored yet by other GWASs. We followed up these genetic correlations to explore whether there are causal relationships (in either direction) between perceived age and genetically correlated traits but did not find strong evidence supporting this. This may reflect the limited statistical power of this follow-up analysis (the genetic correlations were of modest magnitude) or that the genetic overlap between perceived age is due predominantly to biological processes that have underlying relevance for many traits rather than causal pathways between these phenotypes.

Aside from capturing the response to environmental stressors, the analysis may also capture genetic associations with stressors. Factors such as smoking and UV exposure related to geographical location were traditionally considered to be purely environmental and therefore uncorrelated with genotype. However, an increasing body of evidence now points to the heritability of the home environment (Kong et al., 2018) and evidence for correlation between genetic data and both socioeconomic conditions (Tyrrell et al., 2017) and latitude (Haworth et al., 2019) in UK Biobank. Despite the reasons for caution, there was little evidence for inflationary bias in the primary results. The lead single variants show good concordance with previously published findings and appear relevant to dermal protection functions, suggesting that the results of the study primarily capture host susceptibility and response to pro-aging stimuli rather than host liability to be exposed to those stimuli.

The existence of genetic predictors of skin function and likely aging trajectory provides opportunities for research and clinical applications. The results may help prioritize relevant biology for a detailed molecular study of photoprotective mechanisms and nominate proteins whose function, if modulated by cosmetic or pharmacological products, might enhance photoprotection. Statistical power for investigations into longitudinal mechanics of skin aging might be boosted by recruiting based on genotype participants who are at greatest risk of accelerated aging. In the longer term, integration of insights from population-level and individual genetic information may pave the way to precision skincare.

Aside from the conceptual limitations of complexity using perceived age as a proxy for skin traits, one practical limitation of this investigation is the use of categorical data, which is cruder than previous approaches such as using a panel of volunteers to guess the age of a participant and compare that with actual age to generate a continuous measure (Liu et al., 2016). As the phenotype used here was self-reported and subjective, there will be some degree of misclassification. We modeled the likely impact of this on statistical power and FDR using simulations. These showed that misclassification in this study likely affected the statistical power and led to some degree of underestimation of effect size at truly associated SNVs but would not lead to false positive associations (Supplementary Text S2, Supplementary Figure S2). Despite these limitations, the study identified association at previously reported positive controls such as variants within *IRF4*, *MC1R*, and *BNC2* with high levels of statistical evidence. This indicates that the large sample size was sufficient to overcome regression dilution bias introduced by misclassification. Although we believe that the properties of the phenotypic assessment in this study would lead to underreporting of association signals, we have not undertaken replication in an independent sample, which is a limitation of the study.

In conclusion, apparent age is a partially heritable trait. The polygenic association signal and results of gene set analysis suggest that diverse mechanisms act to preserve a youthful appearance. Biological and functional characterization of the single variant association signals identified in this study may be a useful way to gain improved understanding of skin biology or a step toward interventions that moderate the rate of age-related skin changes.

## Materials and Methods

### Participants and phenotypes

This study used data from UK Biobank, a project which recruited approximately 500,000 participants aged between 40–69 between 2006 and 2010 (Fry et al., 2017). Eligible participants were identified from health records in the UK National Health Service and invited to participate in one of 22 assessment centers, which were in densely populated regions of Great Britain. Participants took part in a baseline assessment including completion of questionnaires, physical measurement, donation of biological samples, and consent for subsequent follow-up via linkage to National Health Service records. In the questionnaire, participants were asked to respond to the question “Do people say that you look…?” The possible answers were “Younger than you are,” “Older than you are,” “About your age,” “Do not know,” or “Prefer not to answer.” For this analysis, participants were coded 1 if they reported that they looked younger, 0 if they reported that they looked older, and 0.5 if they reported that they looked their age. Participants who did not know or preferred not to answer the question were excluded from analysis.

### Genotypes

Genotype data was generated using one of two genotyping arrays, the UK BiLEVE Axiom array and the UK Biobank Axiom array. Quality control and imputation were undertaken centrally by UK Biobank as described previously (C Bycroft, unpublished data). Following imputation, in-house quality control was undertaken to remove participants with poor quality data and to restrict analysis to participants of European ancestry, following a published protocol (Mitchell et al., 2017). Genotype data was filtered to a high-confidence set of SNVs by removing monomorphic or rare variants with MAF < 0.1%, removing structural variation such as insertion-deletions, removing sites with poor imputation quality using a graded filter (minimum INFO score > 0.3 for MAF > 3%, INFO > 0.6 for MAF 1–3%, INFO > 0.8 for MAF 0.5–1%, and INFO > 0.9 for MAF 0.1–0.5%) and removing sites not in the Haplotype Reference Consortium imputation panel (Mitchell et al., 2017).

### Genome-wide association analysis

Genome-wide analysis was performed using a linear mixed model approach implemented in BOLT-LMM (Loh et al., 2018). This tests the relationship between genotype and phenotype while accounting for covariates (age, sex, and study participation center) and relatedness, following a published protocol (Elsworth et al., 2017). This approach was chosen because the linear mixed model approach is reported to achieve good control for potential confounding owing to population stratification in the UK Biobank sample (Loh et al., 2018). Genome-wide summary statistics on a linear scale were transformed into log ORs using a Taylor expansion series. ORs > 1 indicate greater odds of looking youthful.

### SNV selection procedure and conditional analysis

SNVs with *P* < 5 × 10^−8^ were considered associated with perceived age, chosen as a threshold for genome-wide significance. Nearby SNVs are typically correlated through LD, meaning that genetic effect sizes and *P*-values of nearby SNVs are also correlated. Lead SNVs were defined after reducing the association signals down to a subset of approximately independent signals of association within single variant results using a stepwise model selection procedure implemented in GCTA (v 1.91.4) (Yang et al., 2012, Yang et al., 2011), which takes into account LD between different SNVs to select independently associated SNVs (--cojo-slct function). This subset of approximately independent signals was tested against previously reported association signals in an approximate conditional analysis (--cojo-cond function in GCTA) to identify which signals capture previously reported associations, using a list of SNVs reported for perceived facial age or related traits (Supplementary Table S9). SNVs that were conditionally independent of previously reported signals of association with *P* < 5 × 10^−8^ in conditional analysis were defined as lead signals and are reported in Supplementary Table S9.

### Enrichment analysis

To test for enrichment in predefined gene sets or gene pathways, enrichment analysis was performed using the DEPICT approach (Pers et al., 2015) implemented in GCT-VL. Analysis used full genome-wide results, and associated loci were defined internally by DEPICT using a reference panel for LD estimation.

### Estimation of heritability

To estimate variation in perceived facial age attributable to common genetic variants, heritability was estimated using univariate LD score regression (Bulik-Sullivan et al., 2015b) implemented in LD-Hub, an automated online resource (Zheng et al., 2017). Summary statistics of the GWAS were uploaded and results processed through a standardized procedure. This uses a subset of approximately 1 million common variants and reference LD data to estimate heritability attributable to common genetic variants (h2__LDSR_) and assess for inflationary bias in GWAS results.

### Estimation of genetic correlation and partial genetic causality

Genetic correlation was assessed against 1,362 traits in the GCT-VL catalog (G Cuéllar-Partida, unpublished data) using bivariate LD score regression (Bulik-Sullivan et al., 2015a). Genetic correlation summarizes the similarity in the heritable contribution to a pair of traits assessed across the whole genome; values near 1 or −1 indicate that two traits have substantial shared genetic associations, which have consistent and proportionate effects on both traits. Values near 0 indicate largely independent genetic determinants with little overlapping heritability between the two traits. Adjustment for multiple testing used a Benjamini-Hochberg procedure and correlations with FDR < 0.05 were reported.

For each trait with a detectable non-zero genetic correlation, latent causal variable models (O’Connor and Price, 2018) were fitted to help distinguish between genetic correlations resulting from horizontally pleiotropic genetic effects and genetic correlations resulting from causal relationships. Models were fitted using an automated online pipeline (S Haworth, unpublished data) implemented in the GCT-VL platform (https://genoma.io).

### Imputed transcriptome-wide association study and transcriptome-informed fine mapping

To test the consequences of a range of gene transcripts on perceived facial age, tests for association with predicted gene expression were performed using S-PrediXcan (Barbeira et al., 2018). This assesses the mediating effects of expression levels on phenotypes by imputing transcriptome levels using pretrained models derived in datasets with measured gene expression. Analysis was performed using prefitted elastic net prediction models of gene expression levels in the 48 Genotype-Tissue Expression tissues (GTEx Consortium et al., 2013), which are available online (http://predictdb.org/). Summary results from GWAS were uploaded to the S-PrediXcan (Barbeira et al., 2018) web pipeline (https://cloud.hakyimlab.org/). Results from 48 tissues were combined using the TissueXcan method (Barbeira et al., 2019), which prioritizes the most relevant tissue transcripts overall, taking into account evidence from multiple tissue-specific predictions while accounting for correlation in gene transcription between different tissues and multiple testing. In parallel, analysis using the FOCUS method (Mancuso et al., 2019) was performed using the standalone python software provided by the authors of the method at https://github.com/bogdanlab/focus/blob/master/README.md. It references LD data from the 1,000 Genomes Project samples (European ancestry) and reference transcription data from the precompiled database, including data from multiple sources described at https://github.com/bogdanlab/focus/wiki.

### Data availability statement

Full results of analysis are provided as supplementary datasets. Underlying source data are available through UK Biobank, an open-access resource for healthcare research. Data access procedures are described at http://www.ukbiobank.ac.uk/wp-content/uploads/2012/09/Access-Procedures-2011-1.pdf. Genome-wide summary statistics have been uploaded to the University of Bristol data repository, data.bris, and are publicly available at https://doi.org/10.5523/bris.21crwsnj4xwjm2g4qi8chathha.

## Ethics Statements

Participants in UK Biobank gave written informed consent and a copy of the consent form is published online at http://www.ukbiobank.ac.uk/wp-content/uploads/2011/06/Consent_form.pdf. The UK Biobank study received ethical approval from the North West Multi-centre Research Ethics Committee (MREC), which covers the UK, as described at (http://www.ukbiobank.ac.uk/the-ethics-and-governance-council/).

## ORCIDs

Victoria Roberts: https://orcid.org/0000-0001-7540-1579

Barry Main: https://orcid.org/0000-0003-0622-805X

Nicholas J. Timpson: https://orcid.org/0000-0002-7141-9189

Simon Haworth: https://orcid.org/0000-0001-7793-7326

## Conflict of Interest

The authors state no conflict of interest.
