# Supplementary material for: Genome-Wide Association Study Identifies Genetic Associations with Perceived Age
Source: J Invest Dermatol. 2020 Dec;140(12):2380–5. doi: 10.1016/j.jid.2020.03.970 (PMC7685007; doi:10.1016/j.jid.2020.03.970)
Supplement: Supplementary Material [file mmc1.pdf]

SUPPLEMENT TO "GENOME WIDE ASSOCIATION STUDY IDENTIFIES GENETIC ASSOCIATIONS WITH PERCEIVED AGE."

V. Roberts, B. Main, N.J. Timpson, S. Haworth

Contents

Supplementary Table 1

Supplementary Table 9

Supplementary Text 1

Supplementary Text 2

Supplementary Figure 1

Supplementary Figure 2

Supplementary Figure 3a

Supplementary Figure 3b

Supplementary Figure 3c

Supplementary Figure 3d

Supplementary Figure 3e

Supplementary Figure 3f

Supplementary Figure 3g

Supplementary Figure 3h

Supplementary Figure 3i

References

Supplementary Table 1: Demographic characteristics of the final sample

	N (% of group) Older than their age	N (% of group) about their age)	N (% of group) younger than their age	Total N (% of study)
Age <45	1,861 (3.5)	14,626 (27.6)	36,457 (68.9)	52,944 (12.5)
45 >= Age < 55	3,262 (2.7)	29,651 (24.1)	89,966 (73.2)	122,879 (29.0)
55 >= Age < 65	2,952 (1.6)	44,629 (24.0)	138,483 (74.4)	186,064 (43.9)
65 >= Age	555 (0.9)	14,394 (23.2)	47,156 (75.9)	62,105 (14.5)
Male sex	6713 (3.5)	54,831 (28.2)	132,847 (68.3)	194,391 (45.8)
Female sex	1917 (0.8)	48469 (21.1)	179215 (78.1)	229,601 (54.2)
Total N (% of study)	8,630 (2.0)	103,300 (24.4)	312,062 (73.6)	423,992 (100.0)

Supplementary Table 9: Previously reported single variant association signals for related traits

RSID	Chr	Pos	Reported trait	P value	Publication
rs12203592	6	396321	Odds of increased pigmented spot severity	1.9×10^{-27}	(Jacobs et al., 2015)
rs35063026	16	89736157		9.4×10^{-15}	
rs6059655	20	32665748		2.6×10^{-9}	
rs12203592	6	396321	Odds of increased skin ageing	8.8×10^{-13}	(Law et al., 2017)
rs1805007	16	89986117		1.2×10^{-10}	
rs4268748	16	90026512		1.2×10^{-15}	
rs185146	5	33952106		4.1×10^{-9}	
rs4911414	20	32729444	Odds of increased tanning ability	3.8×10^{-9}	(Zhang et al., 2013)
rs12913832	15	28365618		1.4×10^{-22}	
rs12203592	6	396321		3.3×10^{-23}	
rs1805007	16	89986117		1.1×10^{-65}	
rs1805008	16	89986144		1.3×10^{-13}	
rs1805007	16	89986117		1.5×10^{-19}	
rs1126809	11	89017961		5.0×10^{-21}	
rs1308048	1	66888542	Odds for category of tanning response	2.1×10^{-14}	(Visconti et al., 2018)
rs12078075	1	2.05E+08		4.0×10^{-9}	
rs9818780	3	1.56E+08		3.4×10^{-8}	
rs16891982	5	33951693		2.0×10^{-176}	
rs251464	5	1.49E+08		2.2×10^{-9}	
rs12203592	6	396321		1.1×10^{-581}	
rs117132860	7	17134708		7.6×10^{-23}	
rs2737212	8	1.17E+08		4.3×10^{-25}	
rs1326797	9	12716762		1.2×10^{-17}	
rs10810650	9	16873551		2.4×10^{-59}	
rs35563099	10	1.2E+08		6.6×10^{-24}	
rs72917317	11	68817441		1.0×10^{-29}	
rs1126809	11	89017961		2.4×10^{-172}	
rs9561570	13	95156198		1.4×10^{-9}	
rs1046793	13	1.14E+08		2.0×10^{-18}	
rs746586	14	92775967		7.0×10^{-13}	
rs12913832	15	28365618		6.3×10^{-184}	
rs369230	16	89645437		1.0×10^{-522}	
rs6059655	20	32665748		1.4×10^{-315}	
rs11703668	22	45630335		1.0×10^{-16}	

RSID: Reference SNP cluster ID, Chr: Chromosome, Pos: Position, P value: reported P value in the original publication.

Supplementary Text 1: Estimation of effective sample size.

The number of participants who self-reported each category of the outcome are reported in the main text. As there is an uneven split in the 3 categories the effective sample size will be smaller than the total number of participants. To help understand the impact of this imbalance on statistical power, we estimated the effective sample size of the experiment as follows.

First, we note that in a conventional case control study, the number of cases is given by $N_{full\ cases}*1 + N_{controls}*0$. Because we considered people who looked about their age to have an intermediate phenotype between a full case and full control, we coded them as 0.5. We therefore reasoned that the effective number of cases could be given as $N_{full\ cases}*1 + N_{half\ cases}*0.5 + N_{controls}*0$.

We used the same approach to estimate the effective number of controls, and finally the effective sample size $N_{eff} = 4/(1/N_{cases}+1/N_{ctrls})$ (Willer et al., 2010).

Using this approach, we estimate there were 60,280 effective controls and 363,712 effective cases, giving an overall effective sample size of 206,839 participants.

Supplementary Text 2: Simulations exploring the potential impact of measurement error

Methods

Simulations were undertaken using the 'simulateGP' package (https://github.com/explodecomputer/simulateGP) in the statistical language R (version 3.5.3, 2019 release). First, 100 genotypes were simulated with minor allele frequency of 0.3 for 423,992 participants. Of these genotypes, 50 had a causal effect on the simulated phenotype, and 50 had no effect. Participant age was simulated from a rectangular distribution between ages 30 and 71. Next, an underlying continuously distributed phenotype was generated representing liability for youthful appearance, affected by both participant age (explaining 50% of variation in the phenotype), the 50 causally related SNPs (explaining collectively 10% of variation in the phenotype) and randomly distributed unmeasured environmental and genetic factors (explaining 40% of variation in the phenotype). Next, underlying liability was altered with the addition of between 0% and 90% random error in 10% increments, i.e. up to 90% of variation in the latent liability variable was now due to additional random noise. At each threshold of noise, the latent phenotype was used to derive a new categorical phenotype with 8,630/103,300/312,062 participants in the respective categories. Next, each derived categorical phenotype was regressed on each causal and non-casual genotype using a linear regression model incorporating adjustment for age. The resulting estimates of genetic effect were flipped where necessary, so the estimates were always positively signed, and then converted to a log odds ratio using the same Taylor expansion series used in the main analysis.

Results

With increasing measurement error, the estimates of genetic effect at truly associated variants are biased towards the null, with an approximately log-linear relationship between odds ratio and percentage of measurement error. With finite statistical power, this means the association with some variants which was detectable in the baseline model is no longer detectable, and the type II error rate of the experiment therefore increases with increasing measurement error. Conversely, the type I error rate is not inflated by this form of measurement error (Supplementary Figure 2).

Supplementary Figure 1: Quantile-Quantile plot of p values from S-PrediXcan analysis.

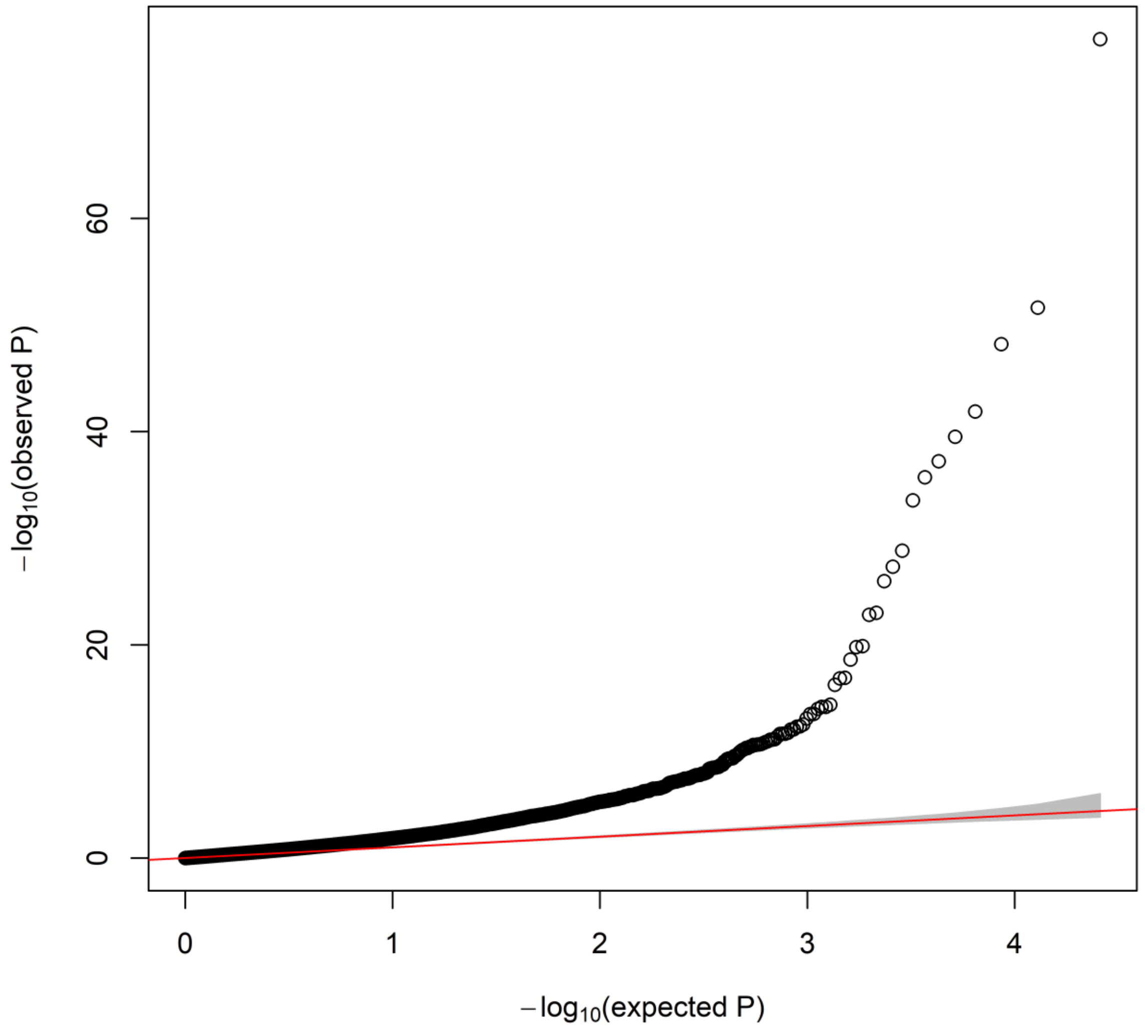

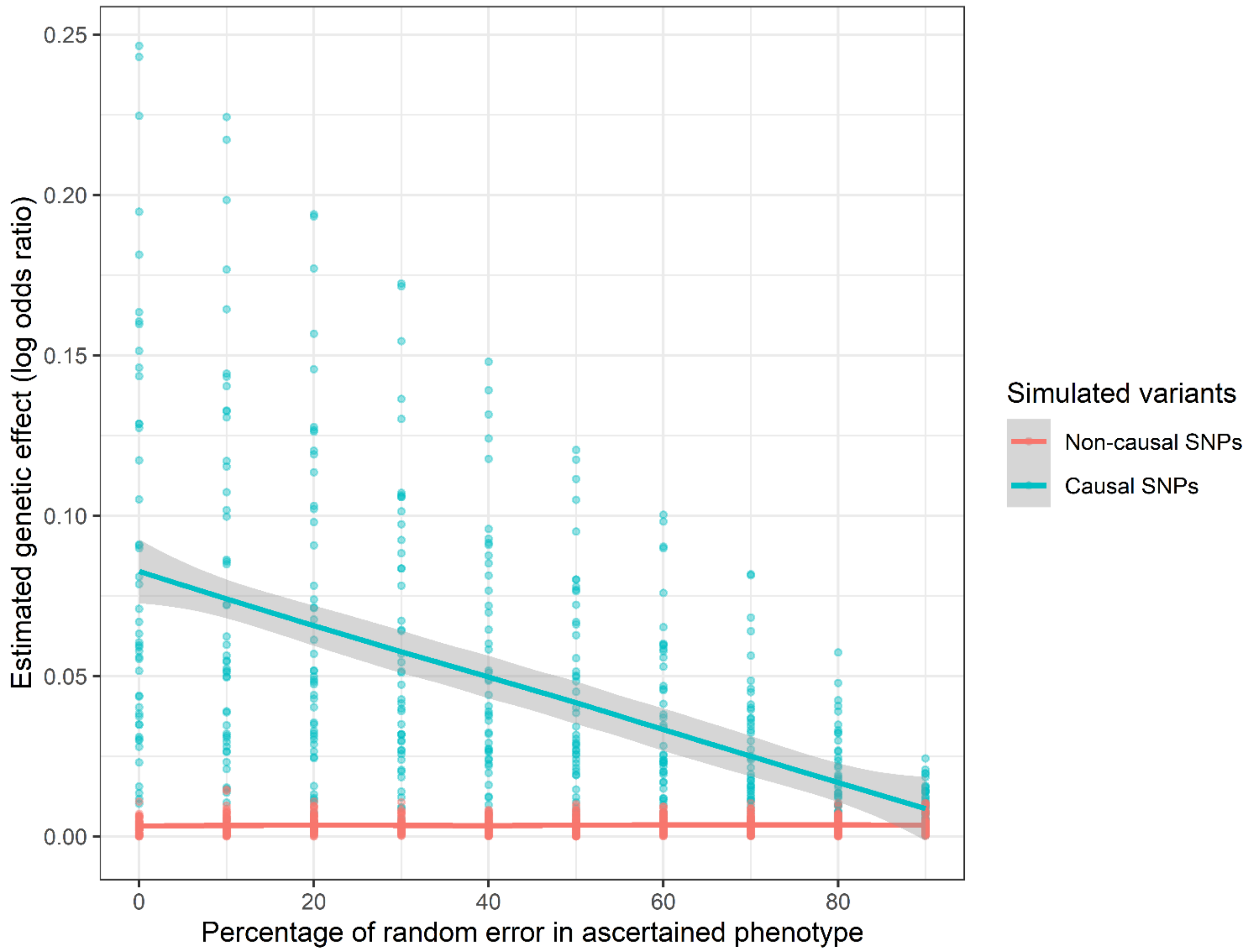

Supplementary Figure 2: Attenuation of apparent genetic effect with increasing measurement error. At each level of measurement error (x axis) there are 100 dots, each representing the association on a log-odds ratio scale between a single simulated genetic variant and self-reported simulated perceived age. The true effect of each SNP is shown in the baseline model (x=0). Variants which have a detectable non-null effect in the true model are colored in cyan, and variants which have a null association in the true model are colored in orange. With increasing measurement error there is a log-linear attenuation in effect sizes away from the true effect (represented by x=0) towards the null for variants with a true effect (cyan regression line), meaning the true associations are no longer detectable for some variants. With increasing measurement error there is no inflation in effect sizes at truly null variants (orange regression line).

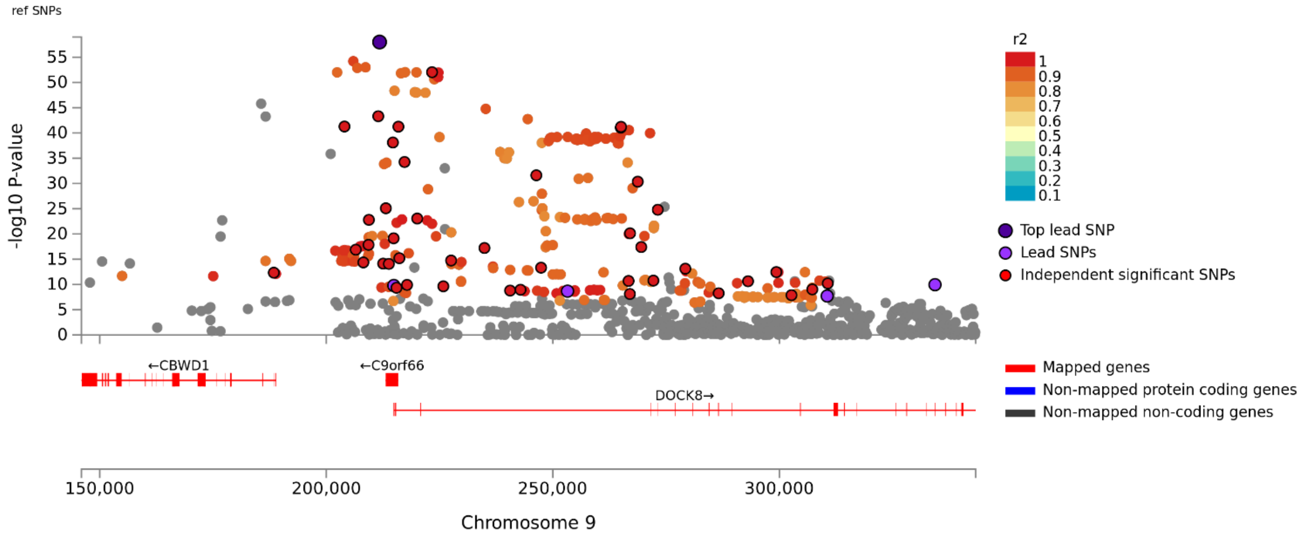

Supplementary Figure 3a: Regional association plot of the *C9orf66-DOCK8* locus

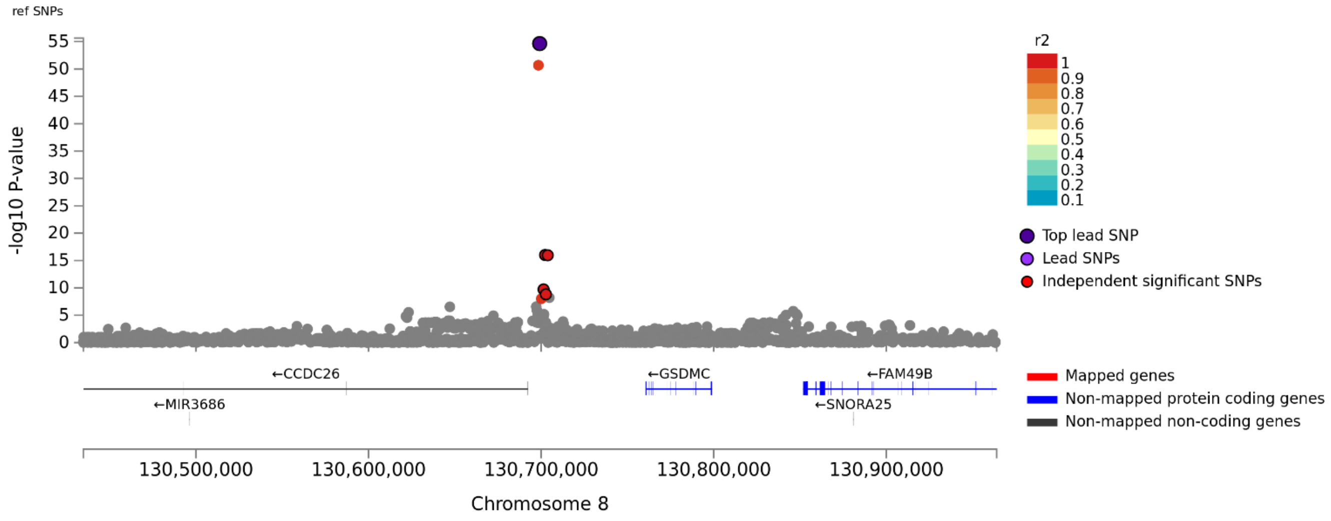

Supplementary Figure 3b: Regional association plot of the *GSDMC* locus

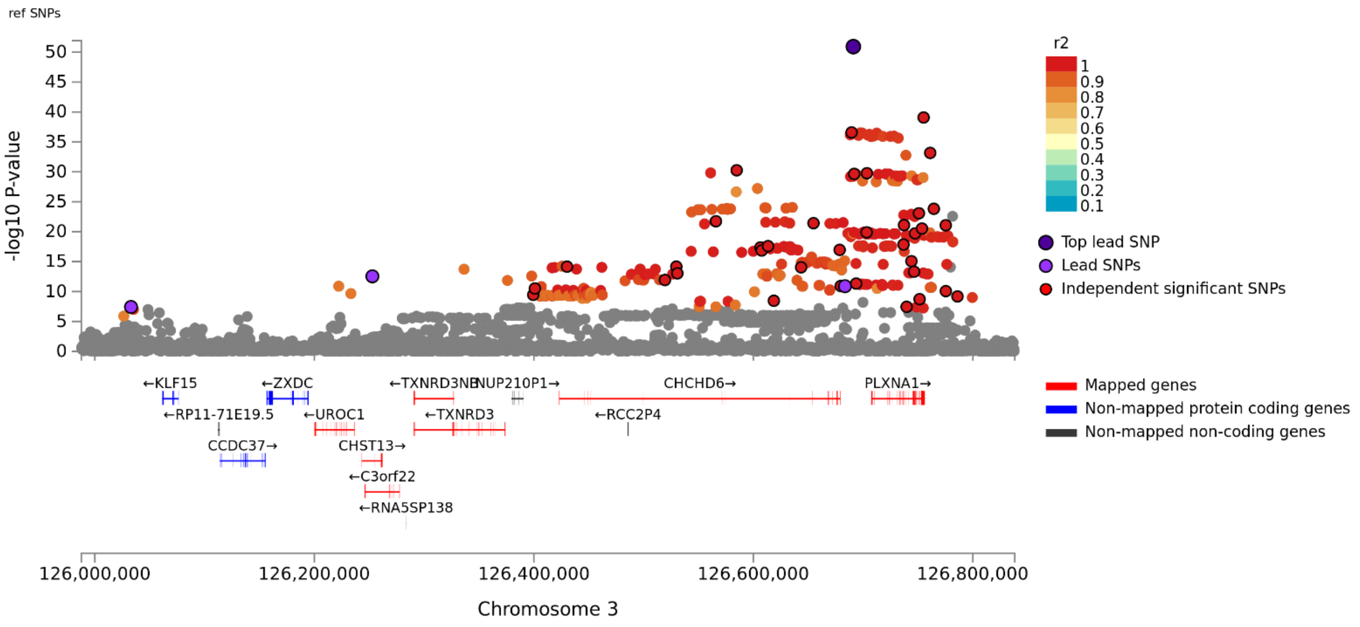

Supplementary Figure 3c: Regional association plot of the *CHCHD6* locus

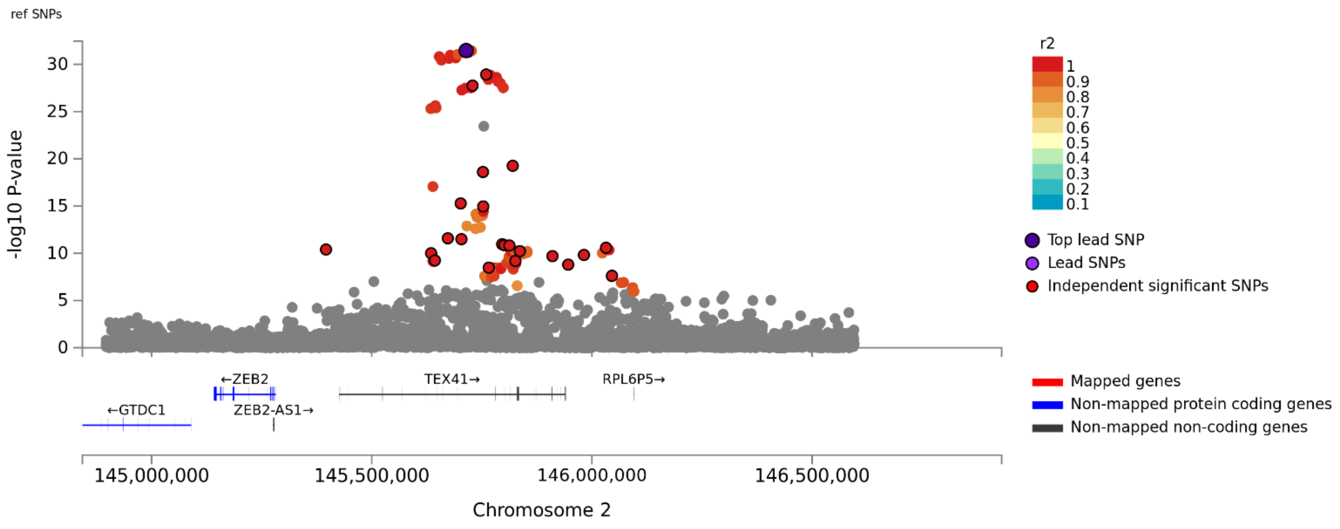

Supplementary Figure 3d: Regional association plot of the *AC074093.1* locus

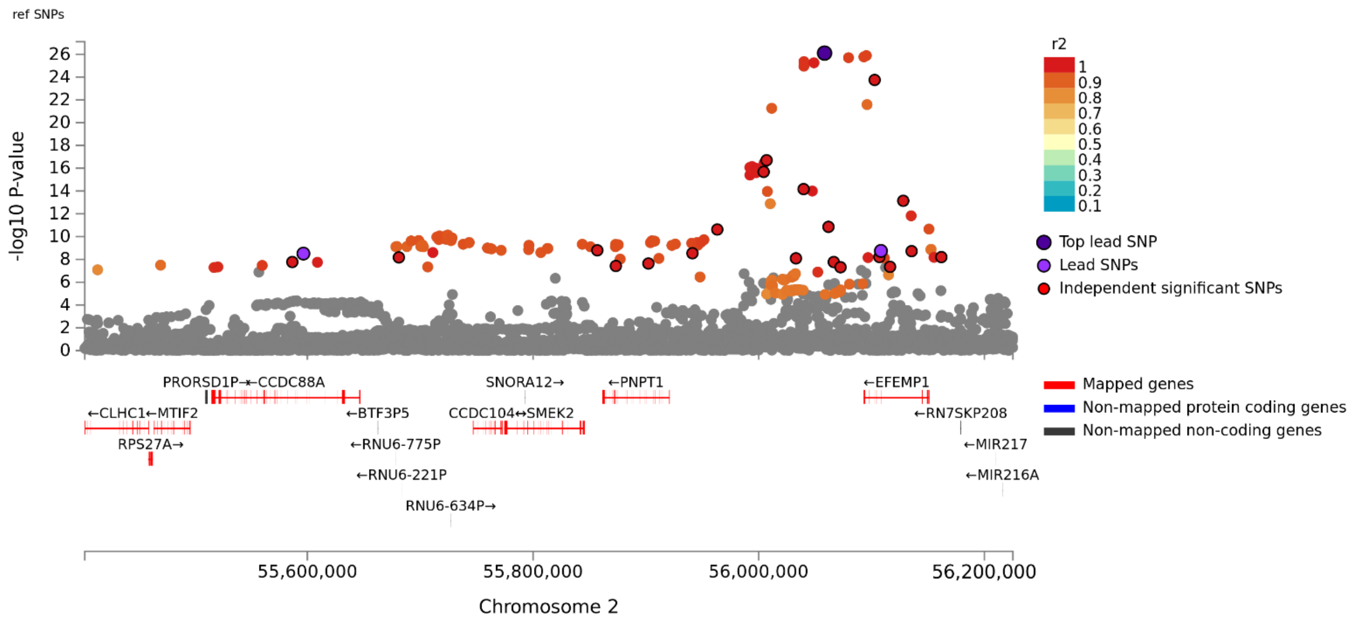

Supplementary Figure 3e: Regional association plot of the *EFEMP1* locus

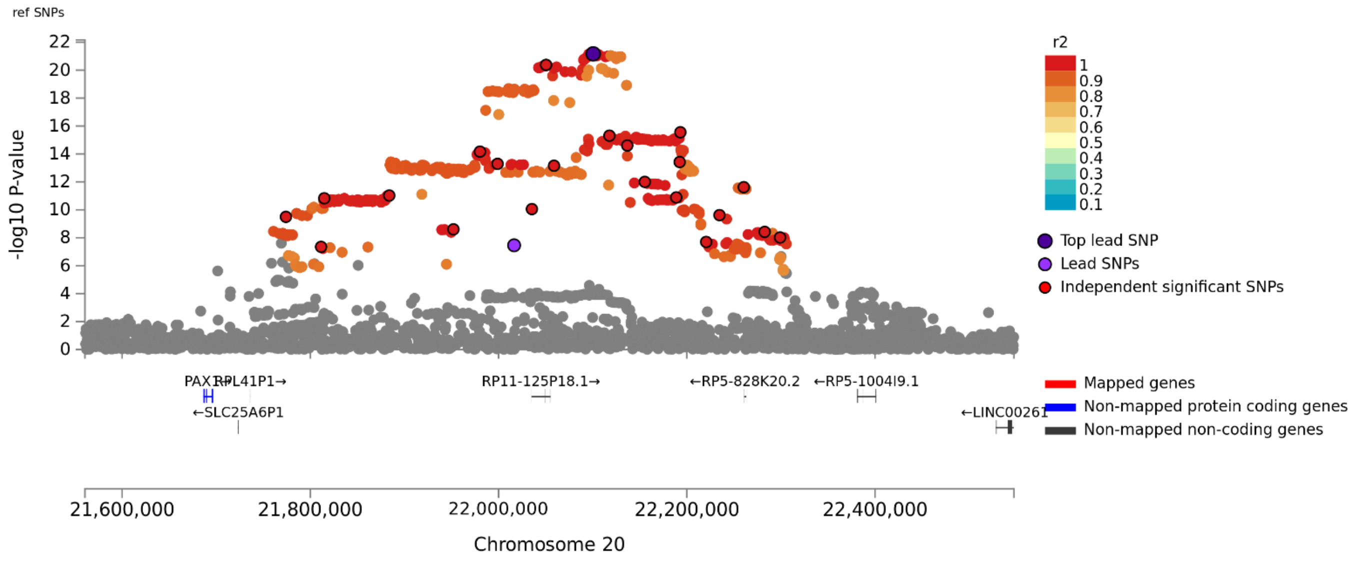

Supplementary Figure 3f: Regional association plot of the *LOC100270679* locus

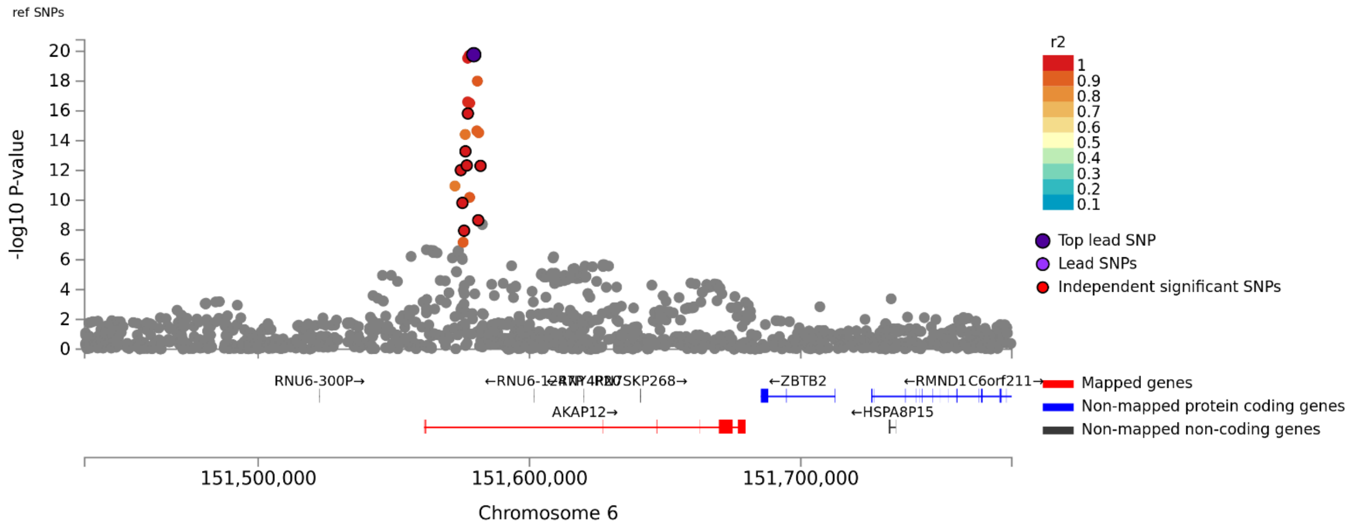

Supplementary Figure 3g: Regional association plot of the *AKAP12* locus

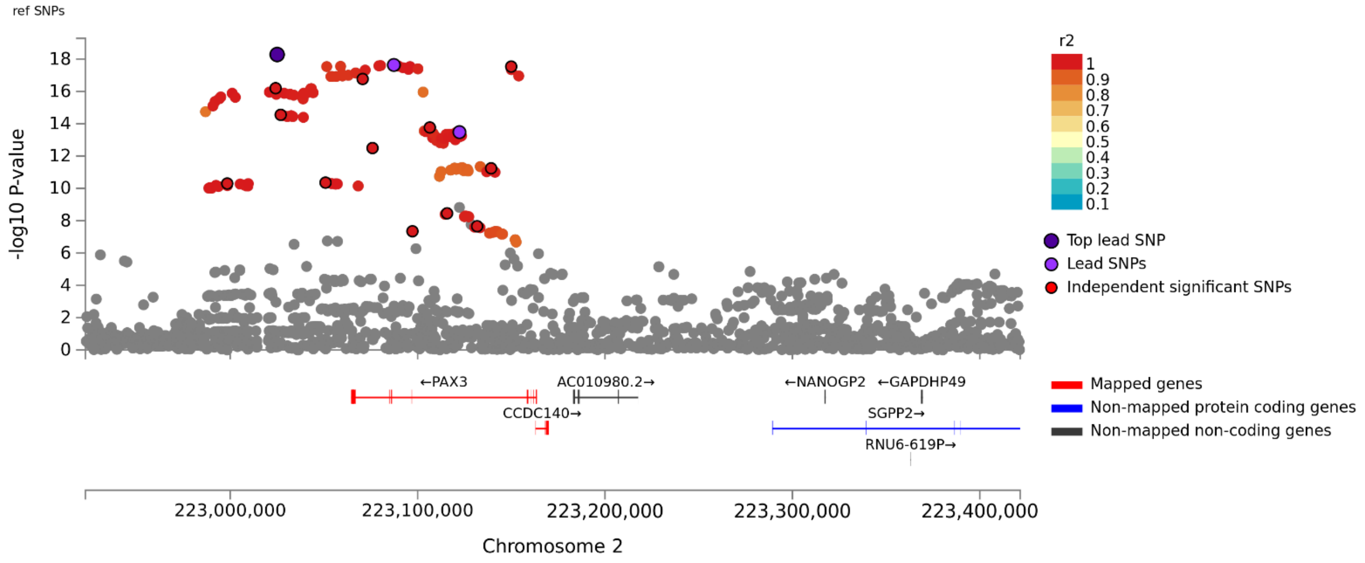

Supplementary Figure 3h: Regional association plot of the *PAX3* locus

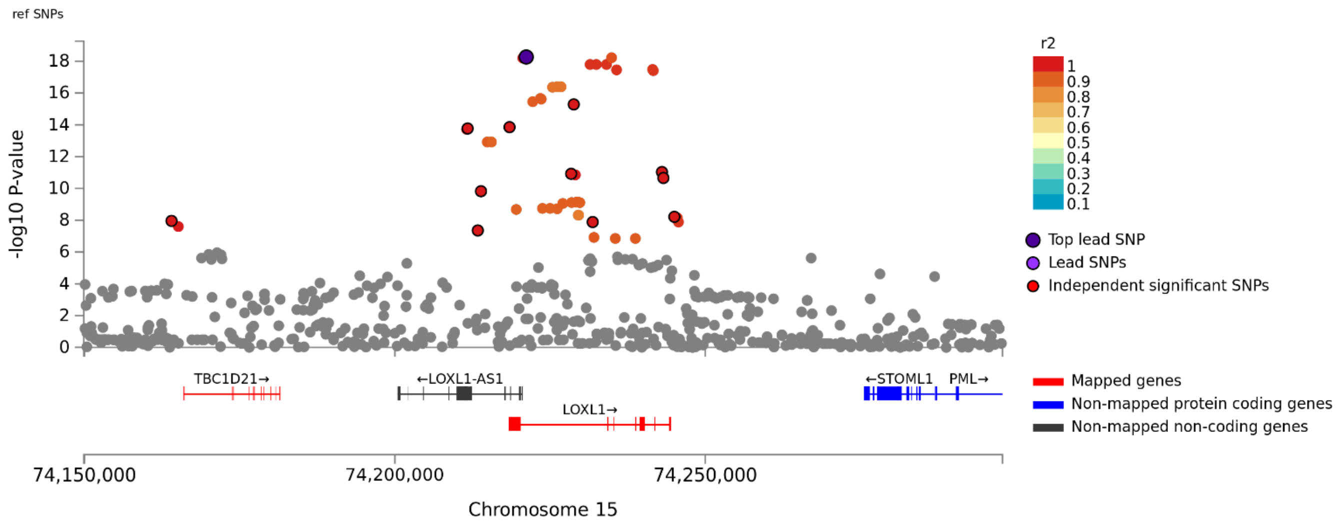

Supplementary Figure 3i: Regional association plot of the *LOX1* locus

References

Jacobs LC, Hamer MA, Gunn DA, Deelen J, Lall JS, van Heemst D, et al. A Genome-Wide Association Study Identifies the Skin Color Genes IRF4, MC1R, ASIP, and BNC2 Influencing Facial Pigmented Spots. Journal of Investigative Dermatology 2015;135(7):1735-42.

Law MH, Medland SE, Zhu G, Yazar S, Viñuela A, Wallace L, et al. Genome-Wide Association Shows that Pigmentation Genes Play a Role in Skin Aging. Journal of Investigative Dermatology 2017;137(9):1887-94.

Visconti A, Duffy DL, Liu F, Zhu G, Wu W, Chen Y, et al. Genome-wide association study in 176,678 Europeans reveals genetic loci for tanning response to sun exposure. Nature Communications 2018;9:1684.

Willer CJ, Li Y, Abecasis GR. METAL: fast and efficient meta-analysis of genomewide association scans. Bioinformatics 2010;26(17):2190-1.

Zhang M, Song F, Liang L, Nan H, Zhang J, Liu H, et al. Genome-wide association studies identify several new loci associated with pigmentation traits and skin cancer risk in European Americans. Human Molecular Genetics 2013;22(14):2948-59.
